# Deep convolutional generative adversarial network for generation of computed tomography images of discontinuously carbon fiber reinforced polymer microstructures

**DOI:** 10.1038/s41598-024-59252-8

**Published:** 2024-04-26

**Authors:** Juliane Blarr, Steffen Klinder, Wilfried V. Liebig, Kaan Inal, Luise Kärger, Kay A. Weidenmann

**Affiliations:** 1 Institute for Applied Materials – Materials Science and Engineering, Karlsruhe Institute of Technology (KIT), Kaiserstraße 12, 76131 Karlsruhe, Baden-Württemberg Germany; 2 Fraunhofer-Institut für Chemische Technologie ICT, Joseph-von-Fraunhofer Straße 7, 76327 Pfinztal, Baden-Württemberg Germany; 3 Mechanical and Mechatronics Engineering, University of Waterloo, 200 University Avenue West, Waterloo, ON N2L 3G1 Canada; 4 Institute of Vehicle Systems Technology (FAST), Karlsruhe Institute of Technology (KIT), Kaiserstraße 12, 76131 Karlsruhe, Baden-Württemberg Germany; 5 Institute of Materials Resource Management, University of Augsburg, Universitätsstraße 2, 86159 Augsburg, Bavaria Germany

**Keywords:** Deep learning, AI, Low contrast, Unsupervised learning, Composites, Computational methods, Computational science, Software, Mechanical engineering

## Abstract

Computed tomography images are of utmost importance when characterizing the heterogeneous and complex microstructure of discontinuously fiber reinforced polymers. However, the devices are expensive and the scans are time- and energy-intensive. Through recent advances in generative adversarial networks, the instantaneous generation of endless numbers of images that are representative of the input images and hold physical significance becomes possible. Hence, this work presents a deep convolutional generative adversarial network trained on approximately 30,000 input images from carbon fiber reinforced polyamide 6 computed tomography scans. The challenge lies in the low contrast between the two constituents caused by the close proximity of the density of polyamide 6 and carbon fibers as well as the small fiber diameter compared to the necessary resolution of the images. In addition, the stochastic, heterogeneous microstructure does not follow any logical or predictable rules exacerbating their generation. The quality of the images generated by the trained network of 256 pixel $\times$ 256 pixel was investigated through the Fréchet inception distance and nearest neighbor considerations based on Euclidean distance and structural similarity index measure. Additional visual qualitative assessment ensured the realistic depiction of the complex mixed single fiber and fiber bundle structure alongside flow-related physically feasible positioning of the fibers in the polymer. The authors foresee additionally huge potential in creating three-dimensional representative volume elements typically used in composites homogenization.

## Introduction

Reducing the weight of designs in the transport and energy sector has shown to be highly efficient in reducing emissions. Especially fiber reinforced polymers are used as engineering materials due to their high lightweight potential, hence high density-specific strength and stiffness^1^. Mostly carbon or glass fibers are used with the latter being the low-cost, yet less strengthening option^2^. The use of thermoplastics as matrix material instead of thermosets has been intensively investigated thanks to their recyclability^3^, which, however, is accompanied by a stronger dependence of the mechanical properties on temperature and humidity^4^. While the determination of material behavior is more complex for composites in general, discontinuously reinforced polymers (DicoFRP) prove challenging to characterize with their complex and heterogeneous microstructure compared to continuously reinforced polymers (CoFRP). However, these DicoFRP impress with their simpler processability^5^. Hence, sophisticated characterization methods and descriptive quantities have been developed. Computed tomography (CT) scans and subsequent image analyses are a common non-destructive testing method to determine quantities like fiber volume content^6^, fiber orientation tensors^7,8^ or fiber length distributions^9^ that can be used to model the oftentimes anisotropic material behavior. Especially in the case of carbon fiber reinforcement, characterization through CT imaging is exacerbated due to the small fiber diameter of 5–7 µm, low contrast in comparison to the also carbon based polymer and therefore reduced image quality (average signal-to-noise ratio of all input images of 5.27, calculated by $\mu /\sigma$)^6,8^.

So-called representative volume elements (RVE) are commonly used in homogenization approaches of composite materials^10–12^. Those unit cells, whose material response is ideally representative of the whole composite material, are typically generated through sphere-packing algorithms. Those range from random sequential adsorption (RSA)^13^, the approaches by Lubachevsky and Stillinger^14^ or Torquato and Jiao^15^ up to algorithms of mechanical contraction^16^. However, all of those methods reach their limits in realistically depicting discontinuous microstructures when it comes to materials with higher fiber volume contents of around 25 %, higher aspect ratios above values of approximately 100^17^, fiber curvature and/or mixed single-fiber and bundle structure. The algorithms either need a high amount of iterations to succeed or fail to generate such microstructures in general^17^. All of the above issues occur for the material dealt with in this work, carbon long fiber reinforced polyamide 6 produced in the long-fiber reinforced thermoplastic direct process (LFT-D)^18^ with an experimentally measured fiber volume content of about 23.57 %^6^ and aspect ratios of over 100. There are more recent and improved approaches to these packing algorithms allowing curvature of the fibers^17^ and even the generation of LFT material^19^. However, all of these approaches are for glass fiber reinforced thermoplastics and not depicting the more complex mixed single-fiber and bundle structure, which is why the use of artificial intelligence (AI) options shall be explored in regards to this problem.

So-called generative adversarial networks (GAN) have been introduced by Ian Goodfellow et al. ^20^, allowing for the creation of images similar to the training images. CT scans are expensive in acquisition and running costs. A trained GAN can produce as many artificial CT images as the user needs instantaneously. Additionally to the prospective use in generating representative volume elements, most machine learning algorithms need a high amount of training data. However, the potential of realistic generated CT images certainly goes far beyond material science and representative volume elements, which is why the authors want to emphasize the extraordinary interdisciplinary scope of possibilities. Especially in the medical field, sufficient training images are often a question of accessibility and privacy, which would cease to be a problem with the creation of artificial CT data (cf.^21^). Furthermore, artificial image generation has long since transcended science and holds industrial potential. For example, it could completely replace graphic design in the future, as the company OpenAI impressively demonstrates with the latest version of Dall$\cdot$E 3^22^.

These GANs consist of two separate, competing multilayer perceptrons, one generator and one discriminator network. The first one is provided with a random noise vector and generates images of prescribed dimensions. The discriminator has access to the training images as well as the generated images and decides on whether a given image is real or fake by assigning a value between zero and one. Subsequently, a weight adjustment through backpropagation takes place depending on whether the decision of the discriminator model was correct or false.

This approach^20^ can be mathematically expressed as a two-player minimax game with the following value function *V*(*G*, *D*):

1 $$\begin{aligned} \begin{aligned} \min _{G} \max _{D} V(D,G)&= \mathbb {E}_{{\varvec{x}}\sim p_{data}({\varvec{x}})} [\log D({\varvec{x}})] \\&\quad + \mathbb {E}_{{\varvec{z}}\sim p_{{\varvec{z}}}({\varvec{z}})} [\log (1-D(G({\varvec{z}})))]. \end{aligned} \end{aligned}$$

In this formula, the discriminator is denoted by *D* and the generator by *G* with noise vectors ${\varvec{z}}$ and real data ${\varvec{x}}$. Consequently, $G({\varvec{z}})$ represents a mapping of the Gaussian sampled random noise vector ${\varvec{z}}$ to the data space resulting in the new generated images. The generator aims to achieve a discriminator feedback close to one (tantamount to the image being realistic or close to the training data) and hence it is trained to maximize the probability $D(G({\varvec{z}}))$. In other words, $\log (1-D(G({\varvec{z}})))$ needs to be minimized. The expected value of a specified distribution function is denoted by the expectation operator $\mathbb {E}$. The discriminator, on the other hand, aims to classify the given samples correctly by assigning labels close to one for real data samples and labels close to zero for generated ones. The probability that a sample is picked from the training data set is given by $D({\varvec{x}})$. Hence, the discriminator is trained to maximize the probability of assigning the correct label to both training examples and samples from the Generator.

The 2016 introduced extension towards a Deep Convolutional GAN (DCGAN) including transposed convolutional layers in the generator and convolutional layers in the discriminator network^23^ is especially powerful for unsupervised learning.

GANs and DCGANs have been used in materials science in various fields, like for microstructure inpainting^24^, virtual microstructure design for steels^25^, generally porous microstructures^26^ or porous 3D anode, cathode or sandstone material^27–29^. There are also works based on the so-called StyleGAN developed by NVIDIA^30^, including the microstructure synthesis of aluminum foam and different kinds of stone^31^ or alloys^32^.

However, in the composites community, GANs have gained only limited traction so far. The authors consider GANs to be a huge area of opportunity, especially in the materials group of discontinuously reinforced polymers with low contrast images and highly complex microstructures. In particular, this opens up new possibilities in the development of the RVEs commonly used in composite homogenization and in fast generation of artificial CT images.

## Results

### Generated images

**Figure 1:**
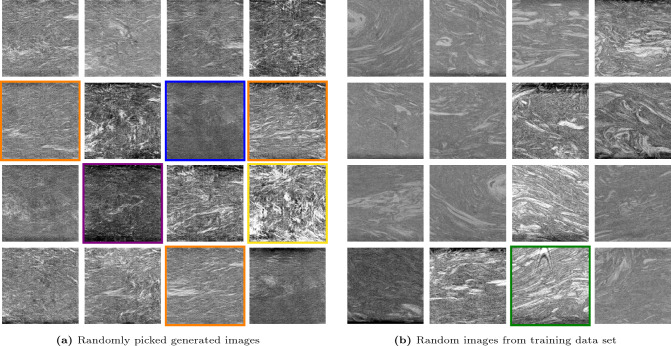
Comparison of (**a**) randomly picked generated images (after 75 training epochs) side by side with (**b**) a random selection of real images of the training data set. Selected images are highlighted in color as they are especially realistic looking (orange), show little to no fibers (blue) or an excessive amount of fibers and fiber bundles (yellow). Furthermore, some real and generated images contain artifacts such as dark image borders (violet) or striped patterns (green).

The DCGAN was trained for 75 epochs on 29,280 input images (cf. “Material and CT scans” section and “Image pre-processing” section) with 128 images per batch corresponding to 228 iterations per epoch when dropping the last non-full batch (cf. “Overview of all chosen parameters” section). A comparison of randomly chosen real and generated images is shown in Fig. 1.

It can be observed that every generated microstructure is different. Generated images look mostly realistic (cf. orange-rimmed images in Fig. 1) including fibers and fiber bundles. The overall fiber orientation resulting from the flow in the compression molding process (cf. “Material and CT scans” section) is visible and the contrast and brightness varies between the images just as in the training images. There are, however, few images which show characteristics that are not represented in the input data set such as two dark border regions on opposite sides of the image (cf. violet-rimmed image in Fig. 1). This phenomenon has to be a result of the combination of features from different images as in the original ones, at most one side displays this artifact related to the scan process. In general, the observed occurrence of dark border regions in the generated images can, for the most part, not be traced back to the DCGAN structure but is subject to the quality of the training data set. On the other hand, it should be positively emphasized that striped patterns (cf. green-rimmed image in Fig. 1), which occur in the training data and represent image artifacts as well, were no longer found in the generated images. As they were not as common as the dark regions on the edge, these artifacts disappeared in the course of the training process. Furthermore, some images might show little to no fibers (cf. blue-rimmed images in Fig. 1) or an excessive amount of fibers and fiber bundles in unrealistic orientations, hence strongly deviating from the general flow direction or showing extreme curvature (cf. yellow-rimmed image in Fig. 1).

### Loss plot

**Figure 2:**
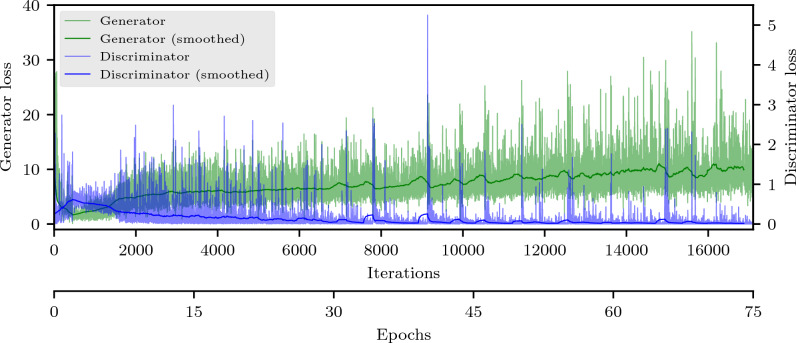
Plot of the generator and discriminator loss of the final network. The smoothed curves of the loss values are calculated as floating average over 228 iterations (corresponds to one epoch). While the discriminator loss increases merely visible at the very start, it decreases to then stay constantly at very low values indicating correct assessment of the images. The generator loss values increase contrarily after a small valley at the beginning but stay roughly at a constant loss range although oscillating heavily.

The loss value of the generator and discriminator network can be analyzed to get information on the stability of the training process. This plot is shown in Fig. 2. It can be seen that both losses approach each other after only a few epochs. However, while the loss of the discriminator stays on a very small value for the rest of the training process, the generator loss increases slightly and oscillations grow bigger. The smallest possible loss values for both generator and discriminator would be zero. However, they cannot simultaneously reach this value. Hence, ideally, both losses should converge to approximately the same value resulting in a balance of generator and discriminator or decrease monotonously on average towards different values indicating a stable training process. This is based on game theory and the aim to reach the Nash equilibrium. Even though the generator loss in Fig. 2 does not decrease monotonously (not taking oscillations into account), the DCGAN in this paper still resulted in a stable training process and the generation of meaningful images. In fact, similar shapes of loss plots have been observed for other GANs as well^33–36^. The behavior of the loss plot and its influence on the quality of the generated images and the need for a different metric to assess them is explained in the Methods (“Methods” section) and Discussion (“Discussion” section).

**Figure 3:**
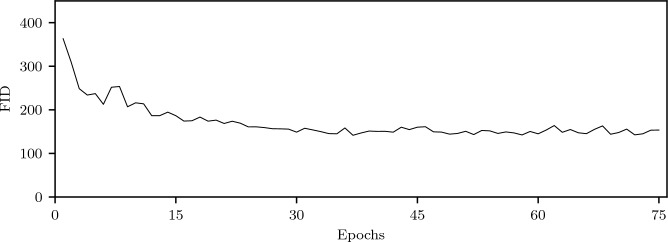
Plot of the FID of images generated by the final network. The values decrease up until about 30 epochs where the distance between the distributions of real and generated images stays more or less constant. The FID hence corresponds more to visual perception than the loss plot, at least in its initial course.

In order to quantitatively judge the generated images even further, the associated Fréchet inception distance (FID) value was calculated after every epoch and is depicted in Fig. 3. It drops sharply during the first few epochs and then remains at this level without major fluctuations. The average of the last 45 epochs is approximately 150.6 with a standard deviation of about 5.7.

This coincides with the visual perception much better than the loss plot: Displaying one randomly chosen generated image based on a fixed noise vector after each epoch (cf. Appendix A, Figure A1 and Appendix B, Figure B1) shows that the images become slightly better over time and not worse, which will be discussed further in “Discussion” section.

### Nearest neighbors

**Figure 4:**
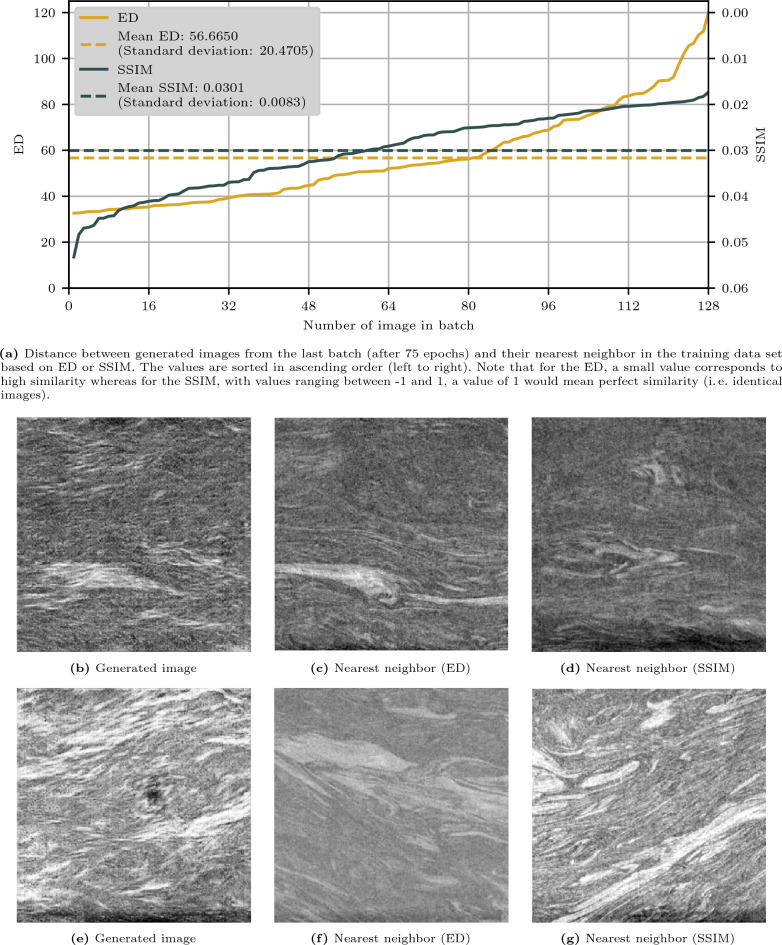
Top: Plot of the ED and SSIM of the generated images from the last batch after 75 epochs and their respective nearest neighbor in the training data set. Bottom: Examples of two generated images (**b**, **e**) and their respective nearest neighbor image of the training data set, based on ED (**c**, **f**) or SSIM (**d**, **g**).

The distance to the nearest neighbor for every image within the last batch of generated images (after 75 epochs) is depicted in Fig. 4a). The values are sorted in ascending order (from left to right). Both for Euclidean distance (ED) and structural similarity index measure (SSIM), there is a wide distribution of images with a relatively similar distance to their nearest neighbor and no noticeable sharp steps in the plot. The range of possible SSIM values is (− 1, 1], meaning that only about 2% is covered in the plot. On the contrary, the ED can output values between 0 and 512 for normalized image tensors of size 256 pixel (px) $\times$ 256 pixel (px) and entries in the range [− 1,1] as used in this work (cf. “Nearest neighbor procedures” Section, Eq. 3). Therefore, the calculated ED values cover a significantly larger proportion of the possible range (more than 16%). However, these values cannot be compared directly since the distribution of distance values is not linear and differs for ED and SSIM. It can be noticed that for the ED the curve rises sharply on the right edge which leads to the assumption that some generated images show a less strong resemblance to even the closest image from the input data set. On the other hand, the SSIM curve drops downwards (i. e. to higher values) at the left indicating that there are also few images that are very close to their nearest neighbor from the input data set based on the SSIM. Please note that for both ED and SSIM it is unclear to the authors, what kind of shape of the curve is desired. Both images of high quality (that means realistic looking images that are no copies of the input data set) and images with a close nearest neighbor as a consequence of copied image sections could score an equally low value. Furthermore, a consistently high quality throughout the entire test batch does not necessarily go hand in hand with an even distribution (i. e. a horizontal line). This is due to the differences in images from the input data set. A side by side comparison of two exemplary generated images (b) and (e) and their corresponding nearest neighbors for both ED and SSIM distance measure is shown in Fig. 4. For example image (b), both methods find decent nearest neighbors. The nearest neighbor determined by ED even seems somewhat closer. However, in the case of image (e), the nearest neighbor that is found through the SSIM measurement fits much better. It appears that for images with clearly recognizable and circumscribed fiber bundles that also appear at the same place as in a training image, ED is a suitable measure. As soon as fibers are rearranged in an angle or shifted in respect to the input images or the amount of fibers in the entire image changes leading to large non-aligning areas, the SSIM was found to be the more robust measure.

In order to not only judge the closeness of a final generated image to the training data set, but also the evolution of their proximity throughout training, the authors plotted the smallest ED and SSIM value of every epoch for one fixed generated image. These results can be seen in the Appendix (cf. Figure A2, A3 for the first image and Figure B2, B3 for the second one) directly after their respective image series (Figure A1, B1). In both cases the ED and SSIM curves decline at the beginning and either increase slightly in the end (ED and SSIM for the first image series, Figure. A2, A3) or stay approximately constant (ED and SSIM for the second image series, Figure B2, B3). It is striking that low values, hence high proximity to the nearest neighbor in the training data set, appear for images that show small amounts of fibers and therefore small gray value fluctuations and a somewhat smooth and evenly distributed structure. Images of this kind appear in the training data set but more often images with widely distributed fibers and fiber bundles occur. Hence, generated images with characteristics close to the latter description should lead to at least equally close nearest neighbors.

## Discussion

GANs offer a previously unmatched image generation capability that works exceptionally well for various image types. There are also some promising initial publications for microstructures, as shown in the introduction. For the gray scale CT images of carbon fiber reinforced polyamide 6 used in this work, the application of this type of network proves challenging. The images do not have quite clearly delineated structures compared to faces or numbers, and follow few rules (such as preferred fiber direction from left to right) compared to, for example, porous structures. And yet, the results show great potential, as the final images generated look quite authentic. From wholly realistic looking images, to images that unfavorably combine certain features of multiple images, to images that show either an extreme amount or little fiber, there is a wide variety of generated images, all of which are within a reasonable range. While the signal-to-noise ratio of the output images is with a value of 3.70 a bit lower than that of the input images, it is still significantly above 1, insuring meaningful signals compared to the background noise. The gray value histogram of the output images is a bit flatter than that of the input images and the maximum value is shifted towards darker values (cf. Figure C1). The cause of this histogram change is difficult to assess, as are possible remedial measures. Adding the histogram mean or even the entire gray value distribution for each image as additional input would be an option (see explanation of conditional GAN (cGAN) and InfoGAN below). The generator loss shows oscillations and does not decrease continuously. However, the training still runs sufficiently stably. The authors ascribe this oscillating behavior of the generator loss to the discriminator quickly getting very good and outputting nearly the same probabilities in later epochs. Thus, the generator gets little meaningful feedback. Both vanishing gradients as well as general convergence problems could be possible responsible failure modes. This loss behavior is not uncommon especially for more complex input and output images like microstructures as already mentioned in the results (cf. “Loss plot” section). In general, finding perfect balance between generator and discriminator is utopian as one looks for a saddle point during optimization and not a minimum as in most other network losses^37^. However, as the generator loss does not increase rapidly, the authors think of the network to be in a metastable state. The yet occurring improvement of the images can be seen by plotting the Fréchet inception distance for each epoch. However, there is a plateau after a while, which could indicate that the images do not improve further. Looking at the evolution of a generated image over the epochs (cf. Figure A in Appendix), a stagnation in the last epochs becomes visible there as well. Judging by the example, the generated images show reasonable results already between epoch 60 and 70. Under these circumstances, the earlier termination of the training process could make sense. However, this cannot be generalized. Based on the progress of another generated image in “Appendix B”, the last image at 75 epochs is better or at least not worse than, e.g., the one of epoch 51. Indeed, the structure can still significantly change during the last epochs, which can be ascribed to the oscillations seen in the loss plot. To sum up, the necessary amount of epochs cannot be specified clearly, with the images showing decent results between 50 and 75 epochs and the authors not expecting tremendous improvement compared to the necessary computational effort after that. Considering the rather high FID value, the authors found the FID being massively biased by the number of samples; that is, the fewer samples are used, the larger the score^38^. Hence, the consideration of absolute values and the comparison with different networks seems to be impossible. However, values in this range occur in other papers with good results as well^32^. Examination of nearest neighbors revealed the DCGAN’s ability to create novel structures. Both metrics (ED and SSIM) work well, with SSIM being the better choice for more novel images. The inability of ED to recognize slight displacements of structures as similar, gives SSIM the expected closer proximity to human perception. However, the time, GPU/CPU, and RAM requirements (cf. “Computational resources and software” section) for more comprehensive SSIM calculations justifies using the ED for quick comparisons. To sum up, ED and SSIM are powerful metrics when looking for nearest neighbors of selected images after the training process. However, the authors do not necessarily recommend the use of those distance measures for judging the training process and thus the evolution of a fixed generated image during multiple epochs. Therefore, FID seems to be the better option, as it fits better to the visible image developments.

In order to give the generated images even more realistic structures, a reasonable further development of the unsupervised DCGAN would be the inclusion of additional information to the training images. This could be image information like the Haralick entropy known from the Haralick features^39^ or physical/mechanical quantities like the fiber volume content, fiber orientation tensor or fiber length distribution contained in the image. The authors assume this would improve the generated images by reducing the lack of semantics and image information in the training images compared to typical data sets used in GANs, like the MNIST data set^40^. In literature, a conditional GAN (cGAN) was introduced which allows the input of a label for each training image^41^. This was attempted by the authors by including one value for the Haralick entropy for each image in the training. However, the significance of one value per image is low; determining multiple values per image in a grid would be more useful. However, a cGAN only allows the classification into categories and no addition of continuous, numerical values. Hence the idea of a continuous conditional GAN (CcGAN)^42^) or also a so-called InfoGAN^43^ would be more suitable. The authors therefore recommend developments towards a “continuous conditional DCGAN (CcDCGAN)” in particular as necessary extension to the DCGAN implemented in this work. The microstructure generator implemented in this work can—once trained—instantaneously generate microstructure images at the push of a button. However, the authors’ vision lies in a microstructure generator that can be given a fiber volume content, a fiber orientation tensor, and a fiber length distribution and then provide a microstructure image with these properties. The implemented network in this paper is the first step.

Another long-term needed enhancement is to read and generate 3D microstructure images, in order to not only obtain 2D CT training images for other networks, but to actually generate 3D representative volume elements that can be used in homogenization procedures.

## Conclusion

In this paper, the authors present a deep convolutional generative adversarial network that instantaneously generates unlimited amounts of realistic CT images of microstructures of carbon fiber reinforced polyamide 6. The network runs stably in the tested number of epochs, although the loss plot of the generator does not fall continuously and shows some oscillations. The Fréchet inception distance of the generated images to the training images decreases during the training. It could be shown through the nearest neighbor methods that due to the relatively high resolution, the multiplication of the given training data set and the use of convolutional layers, the network does not only reproduce images from the training data set but can generate independent, realistic structures. The network provided can be used to generate training CT images for other kinds of image-based artificial neural networks. With the help of the provided code and image data, it can be trained further on slightly different material combinations of fiber reinforced polymers or used with entirely different input images like medical CT scans.

## Methods

### Computational resources and software

The training was performed using the high performance computing resources of the bwUniCluster 2.0, more specific four NVIDIA Tesla V100 GPUs with 32 GB accelerator memory each. Two Intel Xeon Gold 6230 processors provided a total of 40 cores at a processor frequency of 2.1 GHz. For the final job 25.36 GB of RAM were used. This entire job ran for about 7 h 5 min. However, the actual training process without initializing modules or any evaluation lasted only 5 h 34 min. The calculation of SSIM values was performed in a subsequent step using no GPU power which took more than 11 h but required only 18 GB of RAM.

The code was entirely written in Python 3.6.8 and is based on the PyTorch 1.10.1 framework^44^ and the Torchvision 0.11.2^45^ library. Numpy 1.19.5^46^, the OpenCV cv2 4.7.0.72^47^ library, Pillow 9.3.0^48^ as well as Matplotlib 3.3.4^49^ were used for image editing respectively creation of plots. Furthermore, some of the image quality measures are based on the TorchMetrics 0.8.2^50^ library and more detailed structure information about the models was obtained by using Torchinfo 1.5.4^51^.

### Material and CT scans

The material used in this work is carbon fiber reinforced polyamide 6. It is produced in the so-called “long fiber reinforced thermoplastic direct process” (LFT-D process)^18^ (cf.^8^ for process diagram). The process is characterized by its inline compounding of the polymer matrix and direct, uncut carbon fiber addition. The fibers are cut due to the shear stress incorporated by the twin extruder.

**Figure 5:**
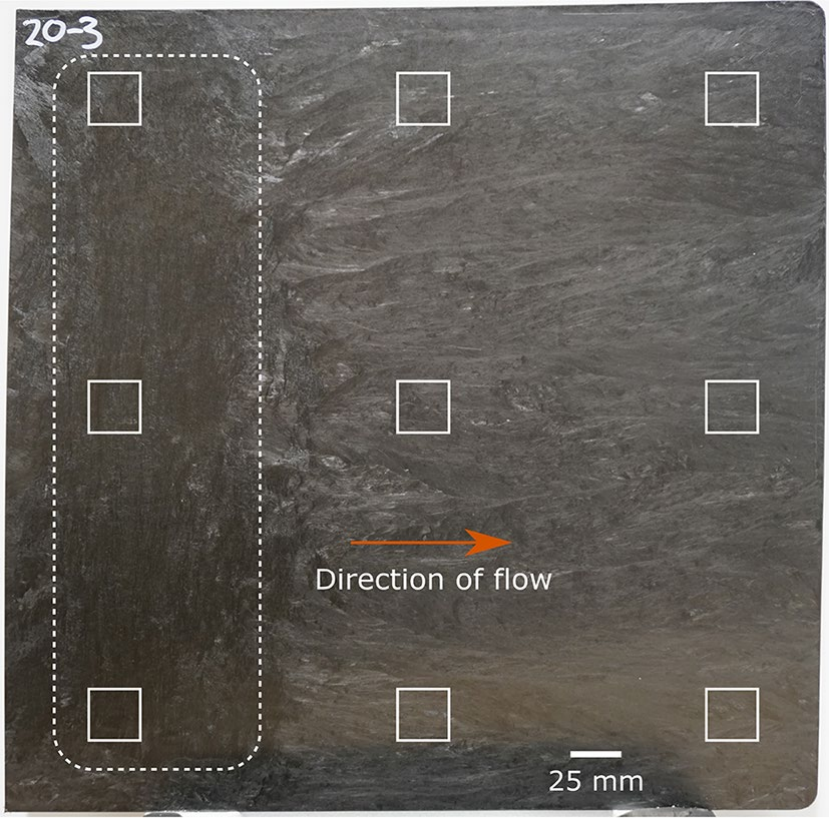
Photograph of a CF-PA6 plaque of size 400 mm $\times$ 400 mm $\times$ 3 mm. The area where the initial charge was inserted is indicated by the white dashed area. The flow direction in the compression molding process was therefore from left to right. The nine white rectangles mark the location and size of the extracted 25 mm $\times$ 25 mm $\times$ 3 mm specimens that were later CT scanned.

The produced plaques were 400 mm $\times$ 400 mm $\times$ 3 mm in size. Nine CT specimens each of two plaques were cut out using waterjet cutting and had dimensions of 25 mm $\times$ 25 mm $\times$ 3 mm with a distance of 50 mm between outer edge of the plaque to outer edge of the extraction area. Three specimens each were taken out of the charge area, the intermediate zone between charge and flow area and the flow area as the microstructures may vary slightly between those regions. The extraction areas can be seen on top of the image of a plaque in Fig. 5.

The specimens were scanned in an YXLON-CT (Yxlon International CT GmbH, Hattingen, Germany) precision µCT system with a µ-focus X-ray transmission tube with tungsten target and a flat panel PerkinElmer (Waltham, MA, USA) Y.XRD1620 detector with 2048 px $\times$ 2048 px. The used scan parameters are given in the following:

- Voltage:    110 kV
- Current:    0.13 mA
- Voxel size:    17.39 µm (plaque 1)
  and 18.06 µm (plaque 2)
- Linebinning parameter:    2
- Number of projections:    2220
- Exposure/Integration time:    800 ms (plaque 1)
  and 1000 ms (plaque 2).

The object rotates 360^∘^ in the beam path in steps of $\varphi = 360^\circ /n_{\textrm{projections}}$ (cf. list before). All of the projections were then reconstructed to a 3D volume by applying the Feldkamp cone-beam algorithm^52^. The final volumes are then sliced perpendicular to the thickness into 2D images which are the basis of the training data. A total of nine specimens each from two identically manufactured CF-PA6 sheets were scanned. The accordingly 18 3D scans had between 155 and 171 slices, i.e. 2D images across the thickness.

### Image pre-processing

The 16 bit (unsigned) raw images from the scans were first converted to 8 bit (unsigned) (256 different gray values). Afterwards, the first and last 30 layers were cut off. There are often edge effects, artifacts and air-rich areas due to the imperfect flatness of the samples in the outer regions. In addition, the outer layers are very low in fibers due to the manufacturing process (cf. shell-core effect of fiber volume content^6^). Even after cutting some layers, enough variety in fiber volume content is given.

In addition to the darker layers at the borders of the thickness (z direction), there are also darker regions at the borders of every slice (x and y direction). Hence, four specific cutout sections of 1024 px $\times$ 1024 px were chosen, each with an arbitrarily set offset of 128 px from the center in x- and y-direction. While chosen arbitrarily as well, the resolution of 1024 px $\times$ 1024 px has the advantage of being a multiple of the later input size of 256 px $\times$ 256 px. This is expected to result in less information getting lost during the interpolation of subsequent resize operations. This cutout procedure quadruples the amount of training data available even before classical augmentation.

Afterwards, the cutout regions were smoothed with a median filter of kernel size 5 px $\times$ 5 px in order to reduce salt-and-pepper noise. The average signal-to-noise ratio of the input images was not deteriorated by this filtering process (5.90 with median filter compared to the value of 5.27 without). The global histogram over the gray value intensities of all images also does not change significantly with or without median filter (cf. Figure C1). Subsequently, each of the images was downsized to 256 px $\times$ 256 px with the “INTER_AREA” interpolation operation from cv2 as a compromise between richness of detail and necessary computing time and power. This results in one pixel having an approximate edge length of about 70 µm.

Following, in order to further increase the amount of input images and therefore potentially get better stability during training, every cutout image is additionally augmented through mirroring on the x- and y-axis and rotation by 180^∘^ (corresponding to a point reflection). Only 180^∘^ rotation was used in order to preserve the overall preferred fiber orientation resulting from the flow in the compression molding process. The entirety of the 29,280 used training images (cf. “Overview of all chosen parameters” section) can be accessed as a data set via the DOI given below.

### Network architecture

**Figure 6:**
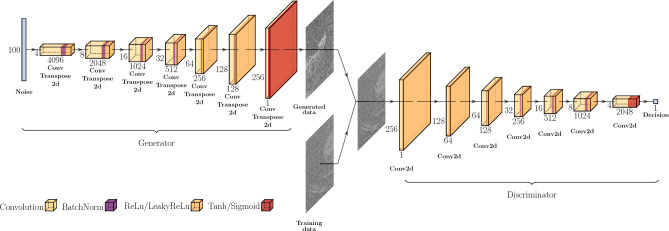
Graphic of the final DCGAN network architecture. Designed with the help of the latex code in the GitHub repository of Haris Iqbal^55^.

The code provided in the official PyTorch tutorial^53^ served as a starting point for the DCGAN structure and was gradually adjusted to work with the given training data set. For larger image resolutions, the DCGAN structure by Milad Hasani^54^ was found to work well. Inspired by this, a network structure for even larger resolutions was developed (cf. Fig. 6). The generator takes a Gaussian sampled noise vector with 100 entries and uses a combination of transposed convolutional layers, batch normalization and ReLU activation to output images with a resolution of 256 px $\times$ 256 px. Tanh was used as final activation. In the discriminator, convolutional layers, batch normalization and Leaky ReLU activations were used. The latter was replaced by a Sigmoid activation in the final layer. The entire code can be found in the GitHub repository linked below.

In a first step, all images from the input data set are loaded as PyTorch tensors (one 3D array per image of x value, y value and gray value) and normalized to values in the range $[-\,1, 1]$ to avoid coefficients equal to 0. The image tensors are then shuffled and divided into individual batches. The last non-full batch is dropped, i. e. the images are not used for training. Unlike in the original DCGAN paper^23^, no initial weights were defined as this was found to increase the likelihood of a stable training process for this particular configuration. The training loss is calculated with the ADAM optimizer based on the commonly used binary cross entropy (BCE). Setting the ADAM optimizer parameters to $(\beta _1, \beta _2) = (0.5, 0.999)$ was found to improve the stability of training just as described by Radford et al.^23^. For multi-GPU parallelization and therefore a faster training NVIDIA CUDA with the “DataParallel” method of PyTorch is used^53^.

The actual training process follows this scheme: First, one batch of generated images is created based on random noise vectors and associated with the corresponding labels (0 for generated). These images are then fed to the discriminator together with one batch containing only real images from the training data set (labeled 1 for real). Based on the average of the calculated losses (following Eq. 1) on both batches, the discriminator biases are updated. As a next step, again Gaussian sampled random noise vectors are passed to the generator which outputs one batch of generated images. Based on the discriminator feedback on these images, the generator is then updated. Every epoch consists of multiple iterations and ends if the whole input data set was processed this way. It shall be mentioned that the losses were saved after every single iteration, whereas the FID was only calculated after every epoch in order to avoid slowing down the training too much. Additionally, after every epoch a number of generated images based on fixed noise vectors was saved in order to visually analyze the training progress later on. Due to the computational effort needed, the image quality assessment was performed in a subsequent step.

### Quantitative quality metrics

Apart from the visual and hence qualitative evaluation of the generated images, multiple quantitative measures have been developed in recent years in order to assess the performance of a generative network^38^. Of those, the Fréchet inception distance (FID) and nearest neighbor evaluations based on two different metrics were conducted that are outlined in the following.

#### Fréchet inception distance (FID)

The Fréchet inception distance^56^, as an advancement of the inception score^57^, is a metric to determine the difference between feature vectors of generated samples and real training images. It is based on the Inception v3 Network^58^ that is pre-trained on the ImageNet^59^. The FID compares the activations from the penultimate layer of the inception network of real $p_r({\varvec{x}})$ and generated $p_g({\varvec{x}})$ images^60^. The distributions of these real and generated images are thereby modeled as multi-dimensional Gaussians that are defined by their mean $\mu$ and covariance *C*. The distance is hence defined by

2 $$\begin{aligned} &d^2 \bigl ( (\mu _r, C_r)(\mu _g, C_g) \bigr ) \\&\quad = ||\mu _r-\mu _g||_2^{2} + tr \bigl (C_r + C_g - 2(C_r C_g)^{1/2} \bigr ). \end{aligned}$$

Thus, a lower FID value corresponds to a smaller distance between the two distributions of real and generated data. The FID values are dependent on the corresponding resize or compression operations and can even improve for higher compressed images (i.e. poorer resolution)^61^. The FID values must therefore be regarded as a benchmark for the quality of images created using the same network in different epochs and are of limited suitability for comparing different networks. The FID was calculated of 128 generated images per epoch.

#### Nearest neighbor procedures

A question that arises quite naturally is whether a network reproduces only the training data, which involves overfitting. To address this, a k-nearest neighbor search is performed, which calculates the k nearest neighbors from the entire training data set to a given generated image. This is equivalent to finding the images in the training data set that have the smallest distance to the generated image based on a suitable distance measure. In the following, we briefly introduce the Euclidean distance and the computationally more expensive structural similarity index measure (SSIM), as these serve as the basis of the k-nearest neighbor search in the results of this paper.

- Euclidean distance (ED)
  The Euclidean distance being one of the simplest ways to determine the similarity between two arrays and the most common distance measure for nearest neighbor search^62^ can be calculated for two gray scale images ${\varvec{x}} = (x_1, x_2, \ldots , x_{MN})$ and ${\varvec{y}} = (y_1, y_2, \ldots , y_{MN})$ of size $M\,\text {px} \times N\,\text {px}$ as:
  3 $$\begin{aligned} d^2_E({\varvec{x}}, {\varvec{y}}) = \sum _{k=1}^{MN} (x_k - y_k)^2 \end{aligned}$$
  with the gray levels at the location (*k*, *l*) given as $x_{kN+l}$ and $y_{kN+l}$. The smaller the calculated value, the higher the similarity. As there are major limitations like the non-consideration of spatial relationships, the nearest neighbors found through the calculation of the ED sometimes do not match human perception. Therefore, a second distance metric is considered. The ED is still calculated due to its computational simplicity.
- Structural similarity index measure (SSIM)
  Another option for the evaluation of nearest neighbors of GANs is the structural similarity index measure (SSIM)^38^ introduced by Wang et al. in 2005^62^. It focuses especially on factors that are also relevant to human perception through evaluating luminance L, contrast C and structure S. Those three aspects are separately mathematically defined through means $\mu _x$ and $\mu _y$, standard deviations $\sigma _x$ and $\sigma _y$ and cross-correlation coefficient $\sigma _{xy}$ of the two images ${\varvec{x}}$ and ${\varvec{y}}$. The detailed formulas can be found in the paper by Wang et al.^62^. In order to avoid instabilities for small values, constants $C_i$ are added. The product of these three quantities relatively weighted through three power parameters $\alpha> 0, \text { } \beta> 0 \text { and } \gamma > 0$ results in the $SSIM({\varvec{x}}, {\varvec{y}})$. Choosing $\alpha = \beta = \gamma = 1 \text { and } C_3 = C_2 / 2$ leads to the following SSIM formula:
  4 $$\begin{aligned} SSIM ( {\varvec{x}}, {\varvec{y}}) = \frac{(2 \mu _x \mu _y + C_1)(2 \sigma _{xy} + C_2)}{(\mu _x^2 + \mu _y^2 + C_1)(\sigma _x^2 + \sigma _y^2 + C_2)}. \end{aligned}$$
  The values range between (− 1, 1] with a value of one corresponding to optimal similarity, hence equality of the images. It shall be mentioned that the SSIM is usually not calculated globally but instead inside of a Gaussian window covering a local square patch which slides pixel by pixel across the entire image. In the so-called mean-SSIM or MSSIM all local values are summed up and divided by the total number of windows *m* in order to obtain one single quality measure. Conventionally, the MSSIM is often referred to as just the SSIM, which will be handled similarly in this work.

### Overview of all chosen parameters

- Image resolution    256 px $\times$ 256 px
- Learning rate    0.0001
- ADAM optimizer    $$(\beta _1, \beta _2) = (0.5, 0.999)$$
- Duration of training    75 epochs
- Number of input images
     $$n_{scans} \times f_{cut} \times f_{augmentation} = n_{final}$$
     $$1,830 \times 4 \times 4 = 29,280$$
- Images per batch    128
- Number of iterations per epoch (the last non-full batch is dropped)
     $$29,280/128 = 228.75$$.

### Supplementary Information

Supplementary Information 1.

## Data Availability

The scans used as data basis for this DCGAN can be accessed through the following DOI: https://doi.org/10.35097/1822.

## Code availability

The complete code of the implemented DCGAN as well as helper functions and accompanying code can be found in the following GitHub repository: https://github.com/sklinder/microDCGAN.
