# Supplementary material for: Deep convolutional generative adversarial network for generation of computed tomography images of discontinuously carbon fiber reinforced polymer microstructures
Source: Sci Rep. 2024 Apr 26;14:9641. doi: 10.1038/s41598-024-59252-8 (PMC11053154; doi:10.1038/s41598-024-59252-8)
Supplement: Supplementary file 1 — Supplementary Information 1. [file 41598_2024_59252_MOESM1_ESM.pdf]

DOI: https://doi.org/10.1038/s41598-024-59252-8

Deep convolutional generative adversarial network for generation of computed tomography images of discontinuously carbon fiber reinforced polymer microstructures

Juliane Blarr[1*], Steffen Klinder[1], Wilfried V. Liebig[1,2], Kaan Inal[3], Luise Kärger[4], Kay A. Weidenmann[5,2]

[1*]Institute for Applied Materials – Materials Science and Engineering, Karlsruhe Institute of Technology (KIT), Kaiserstraße 12, Karlsruhe, 76131, Baden-Württemberg, Germany.
[2]Fraunhofer-Institut für Chemische Technologie ICT, Joseph-von-Fraunhofer Straße 7, Pfinztal, 76327, Baden-Württemberg, Germany.
[3]Mechanical and Mechatronics Engineering, University of Waterloo, 200 University Avenue West, Waterloo, N2L 3G1, Ontario, Canada.
[4]Institute of Vehicle Systems Technology (FAST), Karlsruhe Institute of Technology (KIT), Kaiserstraße 12, Karlsruhe, 76131, Baden-Württemberg, Germany.
[5]Institute of Materials Resource Management, University of Augsburg, Universitätsstraße 2, Augsburg, 86159, Bavaria, Germany.

*Corresponding author(s). E-mail(s): juliane.blarr@kit.edu;
Contributing authors: steffen.klinder@student.kit.edu; wilfried.liebig@kit.edu; kinal@uwaterloo.ca; luise.kaerger@kit.edu; kay.weidenmann@mrm.uni-augsburg.de;

Appendix A Evolution of a generated image (example A) during training

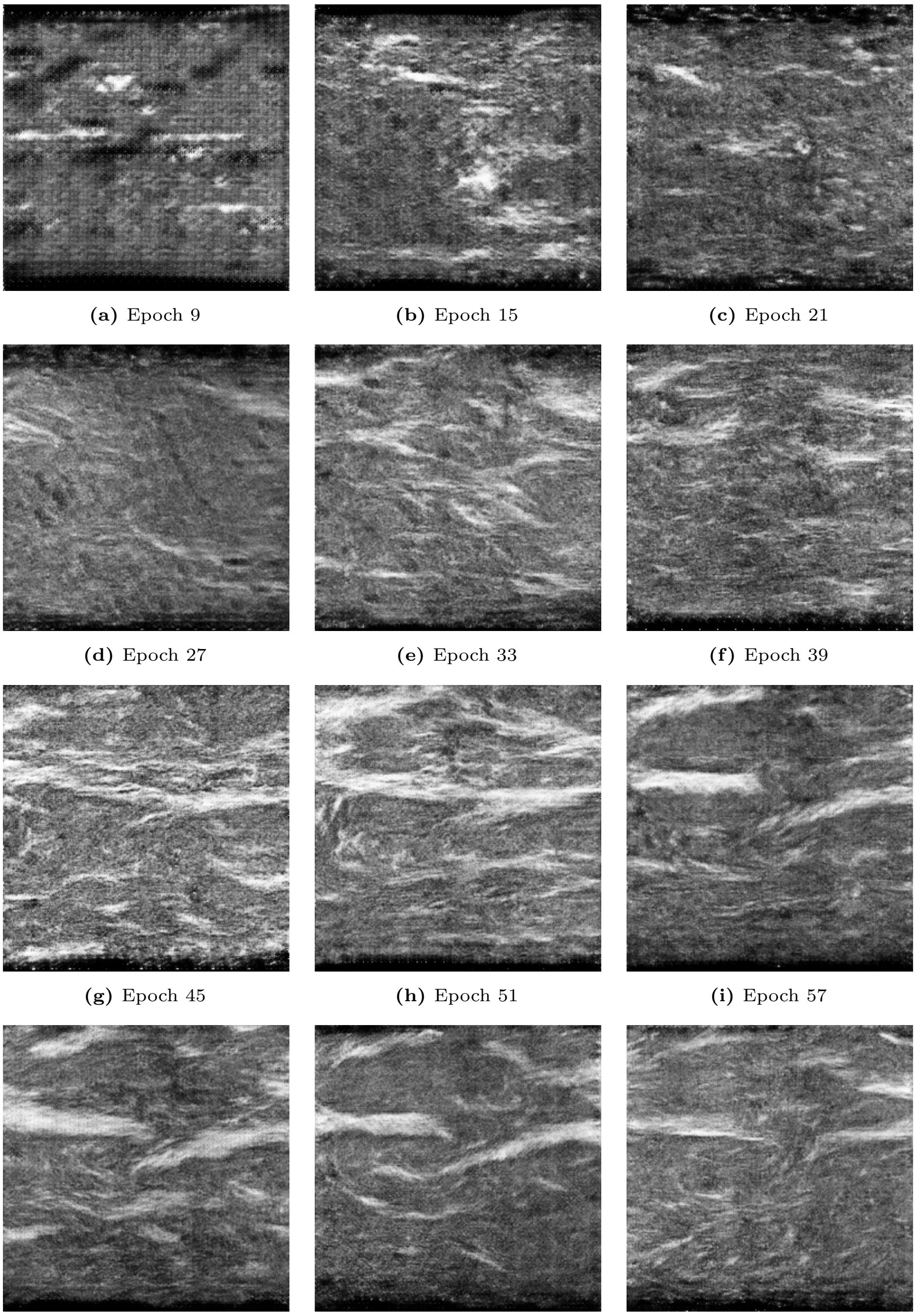

(a) Epoch 9 **(b)** Epoch 15 **(c)** Epoch 21

(d) Epoch 27 **(e)** Epoch 33 **(f)** Epoch 39

(g) Epoch 45 **(h)** Epoch 51 **(i)** Epoch 57

(j) Epoch 63 **(k)** Epoch 69 **(l)** Epoch 75

Fig. A1: Evolution of a selected representative image. From epoch to epoch, new microstructure characteristics emerge and existing features are refined.

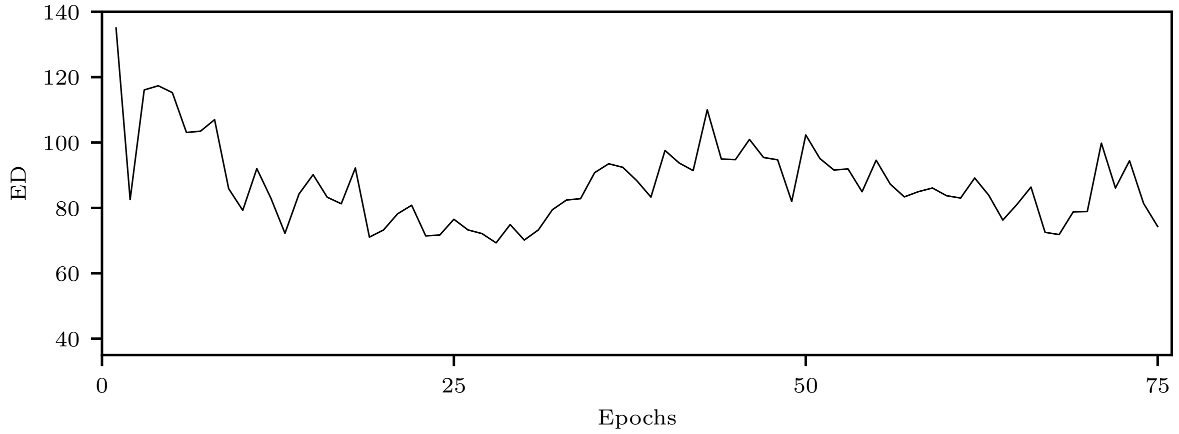

Fig. A2: Plot of the ED between the chosen image and the training data set over the duration of training.

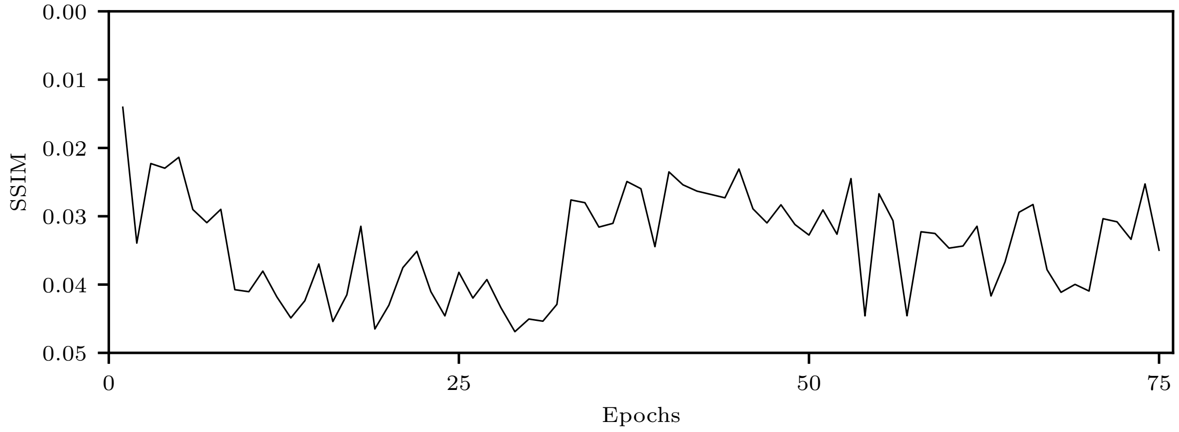

Fig. A3: Plot of the SSIM between the chosen image and the training data set over the duration of training (higher value corresponds to higher similarity).

Appendix B Evolution of a generated image (example B) during training

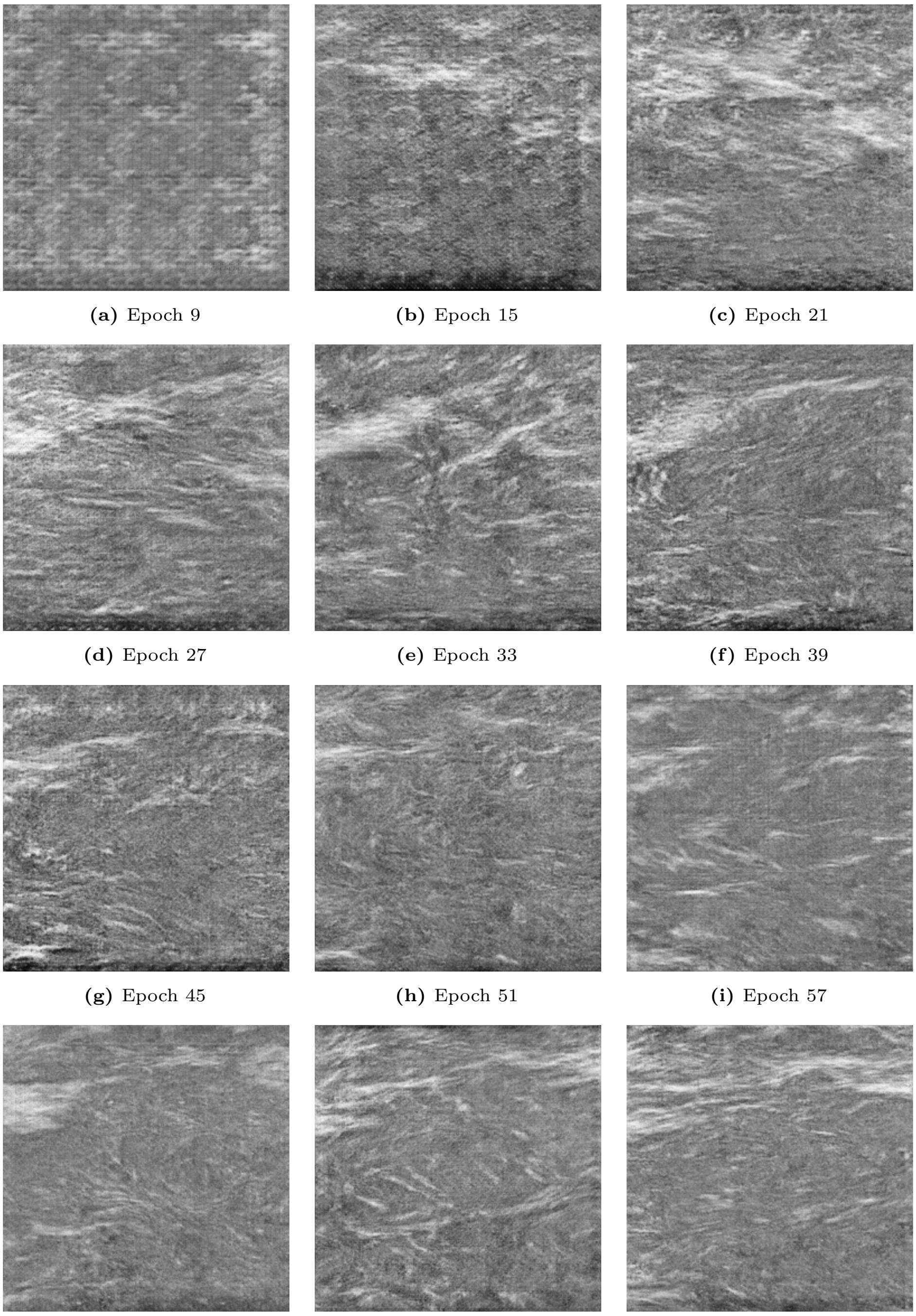

(a) Epoch 9 **(b)** Epoch 15 **(c)** Epoch 21

(d) Epoch 27 **(e)** Epoch 33 **(f)** Epoch 39

(g) Epoch 45 **(h)** Epoch 51 **(i)** Epoch 57

(j) Epoch 63 **(k)** Epoch 69 **(l)** Epoch 75

Fig. B1: Evolution of a selected representative image. From epoch to epoch, new microstructure characteristics emerge and existing features are refined.

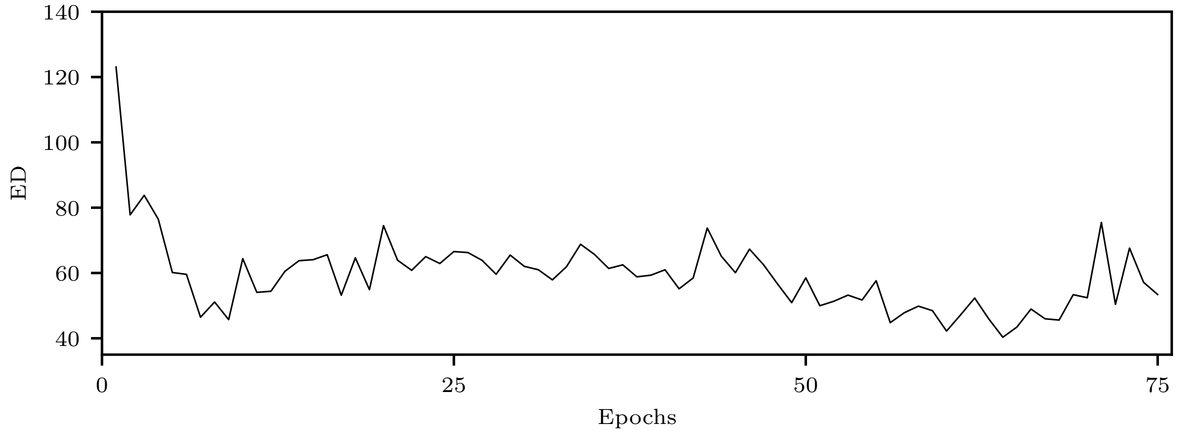

Fig. B2: Plot of the ED between the chosen image and the training data set over the duration of training.

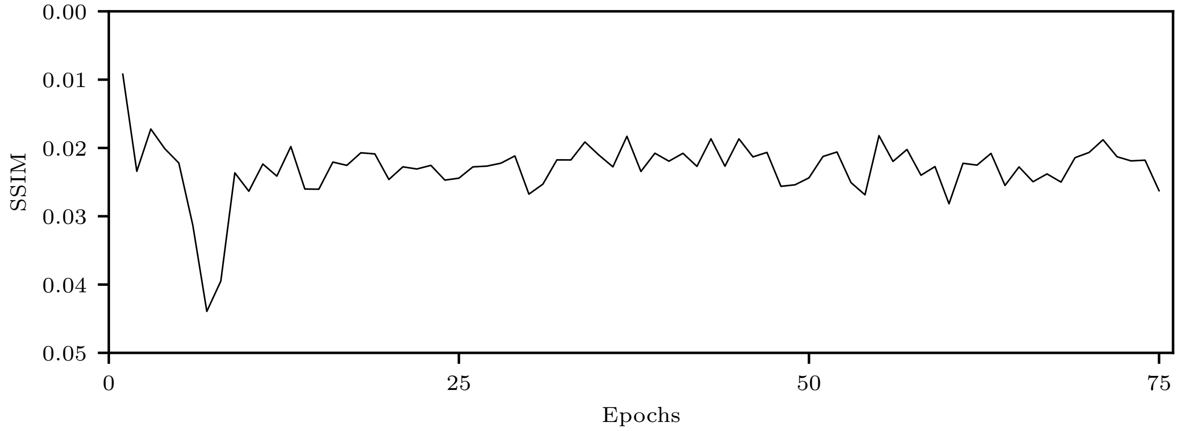

Fig. B3: Plot of the SSIM between the chosen image and the training data set over the duration of training (higher value corresponds to higher similarity).

Appendix C Gray value histograms

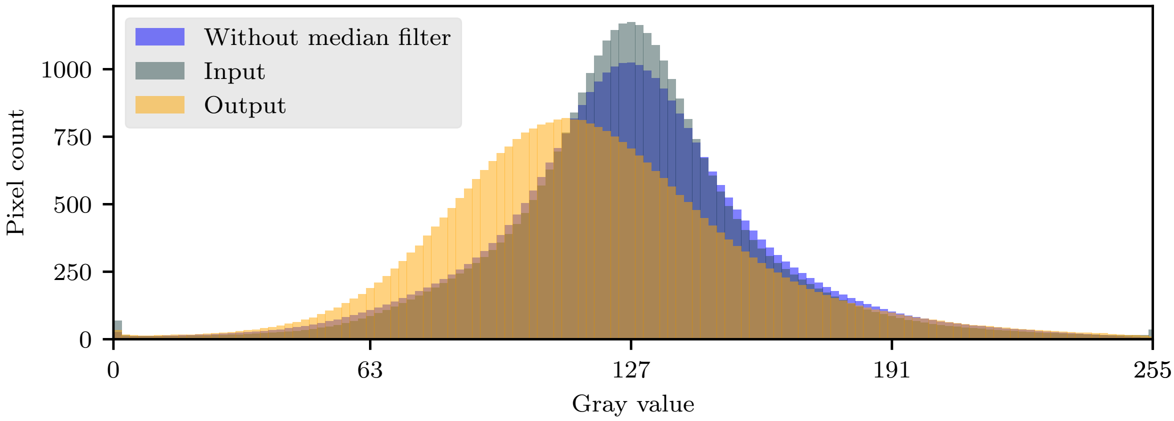

Fig. C1: Global histogram of gray value intensities of all input images without median filter and with median filter as well as of the batch of 128 output images.
